# Pictorial essay: Computed tomography findings in acute aortic syndromes

**DOI:** 10.4102/sajr.v22i1.1309

**Published:** 2018-05-23

**Authors:** Navdeep Singh, Pankaj Goel, Yadwinder Singh

**Affiliations:** 1 Department of Radiology, Ivy Hospital, Amritsar, India; 2 Department of Cardiovascular Surgery, Ivy Hospital, Amritsar, India; 3 Department of Cardiology, Ivy Hospital, Amritsar, India

## Abstract

Acute aortic emergencies are life-threatening conditions that may require urgent surgical or interventional management. Imaging plays an important role in the diagnosis and planning of the management, and timely intervention helps in reducing mortality and morbidity.

## Introduction

Acute aortic syndrome (AAS) is a spectrum of life-threatening aortic diseases consisting of aortic dissection (AD), intramural haematoma (IMH) and penetrating atherosclerotic ulcer (PAU).^1^ Acute aortic syndrome is often part of a large clinical group of patients presenting with acute chest pain to the emergency room and often difficult to distinguish initially from other causes such as myocardial infarction, pulmonary embolism, and so on. Clinical presentation and histological findings of the three entities are similar, typically presenting with acute onset of chest pain and histologically characterised by disruption of the aortic media.^2^ Diagnosis based upon clinical symptoms and physical examination alone is not possible. A multidisciplinary approach is required to reach an accurate diagnosis and for timely intervention.

Multidetector computed tomography (MDCT), transoesophageal echocardiography and magnetic resonance imaging are the imaging modalities which could diagnose AASs. However, computed tomography (CT) is the most important investigation performed in acute emergency situations, owing to its accessibility, rapid acquisition and high sensitivity and specificity. Apart from diagnosing, the purpose of imaging is to locate the exact site, extension of disease, complications and associated findings which may affect the therapeutic management.

## Imaging

Triple-rule-out (TRO) CT angiography may be employed for evaluation of acute chest pain, as it examines the thoracic aorta, and pulmonary and coronary arteries in a single study. It is a useful investigation in low and moderate risk acute coronary syndrome patients; however, performing TRO is technically challenging. The protocol needs to be customised according to the patient profile to achieve the best results. There are studies which did not show any improved clinical outcome of TRO, as compared with dedicated pulmonary and coronary angiographies. Also, a higher radiation exposure and large contrast medium doses are two major disadvantages of TRO.^3^

As the risk and clinical profile of patients with AD and IMH are similar, unenhanced CT is recommended, prior to a contrast study, in patients suspected with AAS and blunt chest trauma. Unenhanced CT acquisition plays a pivotal role in the diagnosis of IMH, as a contrast study alone is not sufficient to make a confident diagnosis. High attenuation contrast decreases the conspicuity to visualise hyperdense IMH. Intimal calcifications and acute haemorrhages, such as haemothorax and mediastinal haematomas are also easily interpreted on unenhanced CT scans.

The recommended scan protocol includes an unenhanced CT scan, followed by a dual phase contrast study, covering the lung apices to the inguinal regions. A total of 100 mL of non-ionic contrast medium containing a high iodine concentration (350 mg/dL) is injected at a flow rate of 4.5 mL/s using an 18-gauge catheter in the right antecubital vein, to reduce artefact from the left brachiocephalic vein. Image acquisition is started using the bolus track technique after placing the region of interest (ROI) on the ascending aorta with the trigger at 200 HU. Automatic acquisition may fail if the ROI is placed over a thrombosed vessel; hence, the technician should be prepared to manually start the study. Electrocardiogram- (ECG-) gated CT is advised when scanning the ascending aorta and arch to reduce motion-related artefacts to produce superior diagnostic-quality images. ECG-based tube current modulation and adjusting the peak voltage according to the patient’s morphology are two of the most effective methods to reduce radiation exposure. In addition, a slow regular heart rate and prospective ECG-gated acquisition could further bring down the dose.

## Aortic dissection

Aortic dissection is the longitudinal splitting of the aortic media when circulating blood enters through a tear in the intima under haemodynamic force, resulting in the formation of true and false lumens. The incidence ranges from 2.6 to 3.5 cases per 100,000 persons per year with a male predominance.^4^ Hypertension is the most common trigger. Marfan syndrome, Ehlers–Danlos syndrome, bicuspid aortic valve, aortic coarctation, Turner syndrome, and cocaine use are considered as some predisposing factors. The ascending aorta near the aortic root is the most common site for dissection initiation and the flap extends along the right anterolateral aspect.^5^ Clinically, AD is characterised by excruciating chest pain radiating to the back. Clinical symptoms also depend upon the extent of the dissection and complications such as ischemia and malperfusion. The DeBakey and Stanford systems used for classification of AD are based on disease location, extension and treatment modalities. Dissection involving the ascending aorta or aortic arch is classified as Stanford type A (DeBakey type I and II) and involvement of the aorta distal to the origin of left subclavian artery is classified as Stanford type B (DeBakey type III). Urgent repair is recommended in type A dissections to avoid lethal complications, whereas type B dissections may be managed conservatively in an uncomplicated patient.^6^

### Imaging findings

**FIGURE 1:**
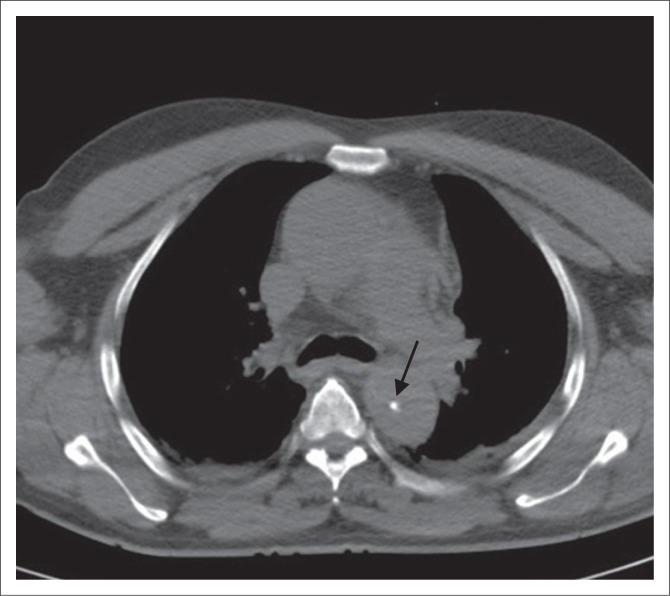
Axial unenhanced computed tomography scan of thorax demonstrating displaced intimal calcification (arrow), suggesting aortic dissection.

**FIGURE 2:**
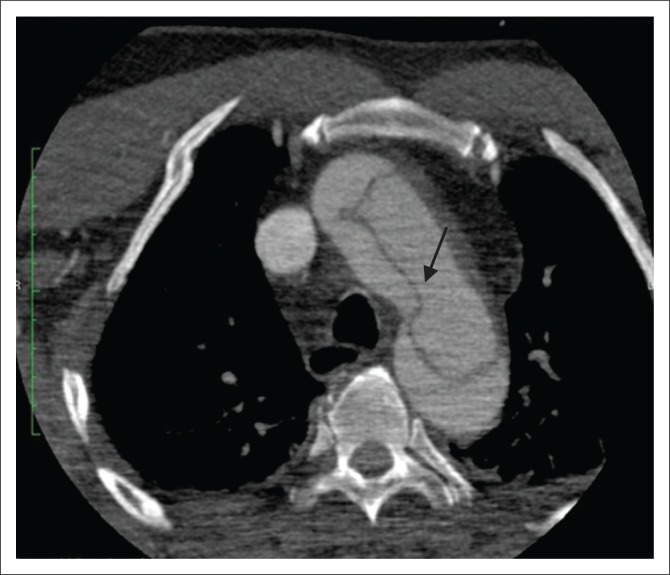
Contrast-enhanced axial computed tomography scan demonstrating a displaced intimal flap (arrow), separating the true and false lumens in a patient with a Stanford Type A aortic dissection.

**FIGURE 3:**
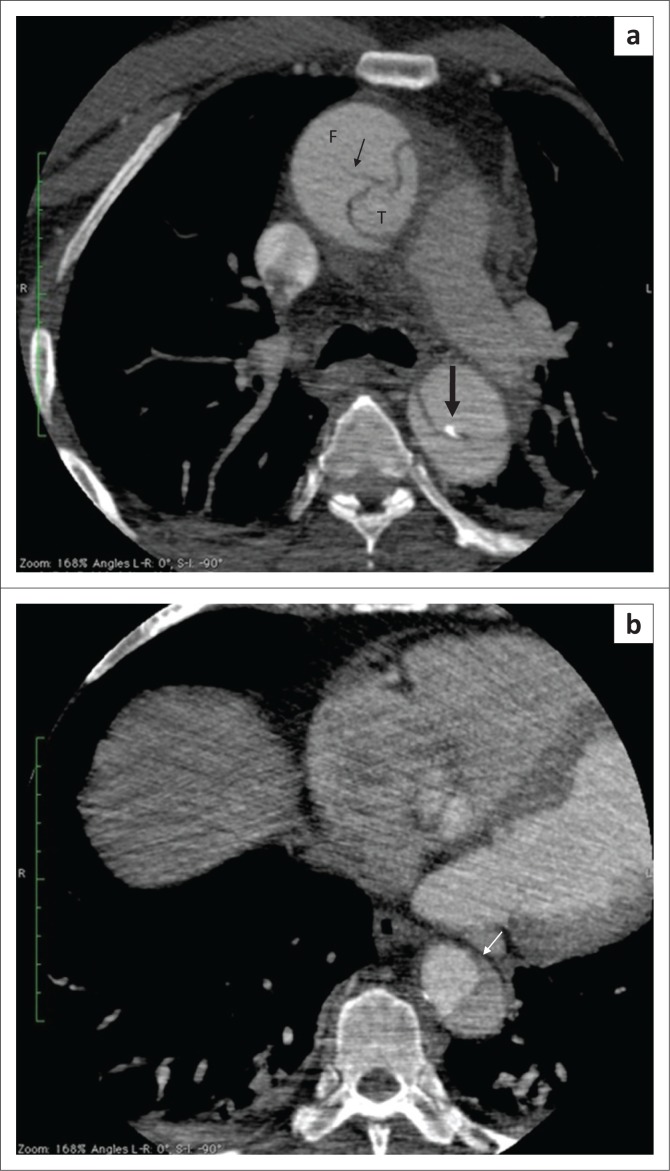
(a) Contrast-enhanced axial computed tomography (CT) scan showing intimal calcifications around the true lumen (thick arrow) and linear scattered hypodense areas within the false lumen termed the Cobweb sign (thin arrow), representing the remnants of media. The calibre of the true lumen (T) appears smaller than the false (F). (b) Axial contrast-enhanced CT scan showing a beak sign (arrow) which is a wedge of haematoma at the distal end of false lumen forming an acute angle with the vessel wall.

**FIGURE 4:**
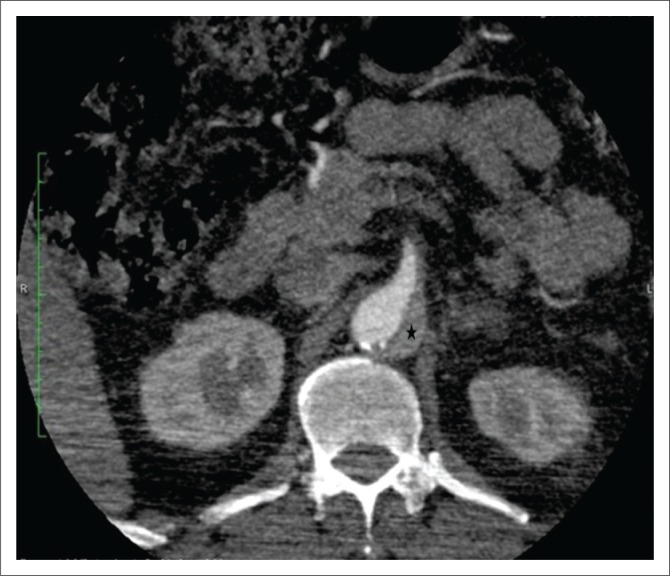
Stanford type B dissection with extension into the superior mesenteric artery. Contrast-enhanced computed tomography showing lesser enhancement in the false lumen (*) as compared to true lumen.

**FIGURE 5:**
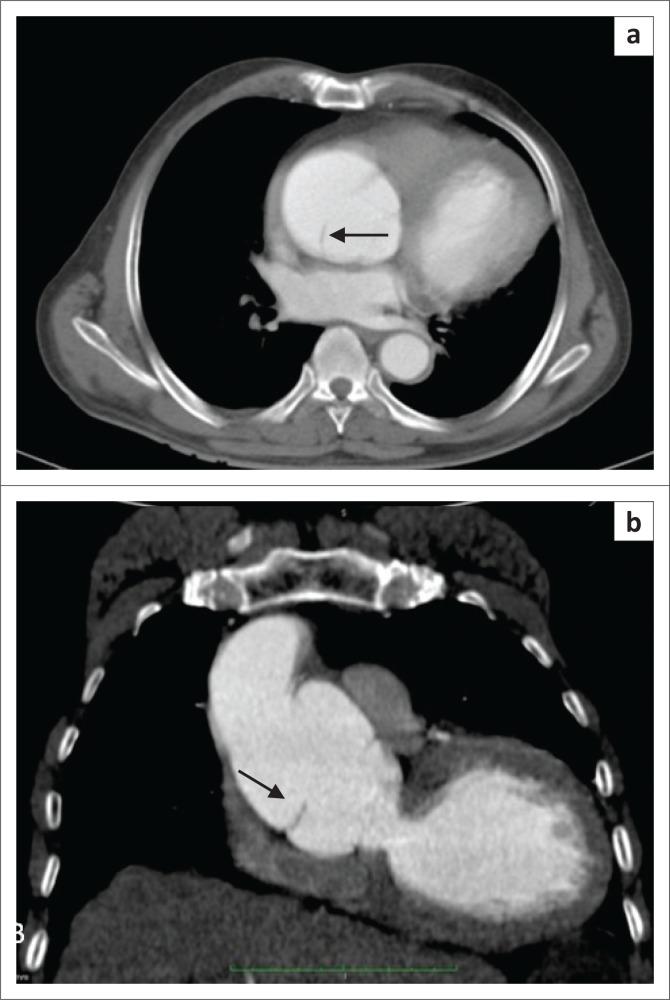
Type A chronic dissection. Contrast-enhanced axial (a) and coronal (b) computed tomography scan showing an ascending aortic aneurysm with chronic dissection. A thin linear low attenuation discontinuous dissection flap (arrow) is seen within the aneurysm.

On unenhanced CT, intraluminal displacement of intimal calcifications can occasionally be seen, suggesting AD (Figure 1). A linear hypodense internally displaced structure representing the intimo-medial flap, separating the true and false lumens, is the most important CT finding seen in approximately 70% of cases (Figures 2, 5 and 8c).^7^ In the present era of endovascular management, differentiation of the true and false lumen is important in planning the management. The true lumen directly communicates with the aorta, and intimal calcifications, if present, surround it (Figure 3a). The calibre of the true lumen is smaller as compared to false lumen, which wedges around it owing to permanent systolic pressure. Fine linear scattered hypodense areas within the false lumen are termed the Cobweb sign (Figure 3a). It is specific for the false lumen and represents collagenous residual media fibres.^8^ The beak sign is another useful sign for the false lumen which represents a wedge of haematoma at the distal end of the false lumen on cross-sectional imaging (Figure 3b). It forms an acute angle between the dissection flap and vessel wall and represents the site of propagation.^9^ The false lumen shows less contrast enhancement compared to the true lumen in the early arterial phase and may appear hyperdense to the true lumen in the venous phase owing to contrast pooling (Figure 4).

Aortic dissection with a thrombosed false lumen could mimic an aortic aneurysm with intraluminal thrombus. Intimal calcifications are useful in differentiating between them, as they are located in the centre in AD and at periphery in aortic aneurysm with thrombus. The dissection flap in AD has a smooth contour, whereas mural thrombus usually has an irregular internal surface.

## Intramural haematoma

Intramural haematoma is the aortic wall thickening, caused by acute haemorrhage contained within the media. It was widely believed that it occurs owing to spontaneous rupture of the vasa vasorum with an intact intima. However, with recent advances in imaging, intimo-medial tears have been detected, suggesting that IMH has one entry tear with no exit, whereas AD has entry and exit intimo-medial tears into the aortic lumen. Intramural haematoma constitutes 5% – 15% of all cases of AAS.^10^ Acute chest pain is the most common presenting symptom. Non-enhanced CT is very important in diagnosing IMH.

### Imaging findings

**FIGURE 6:**
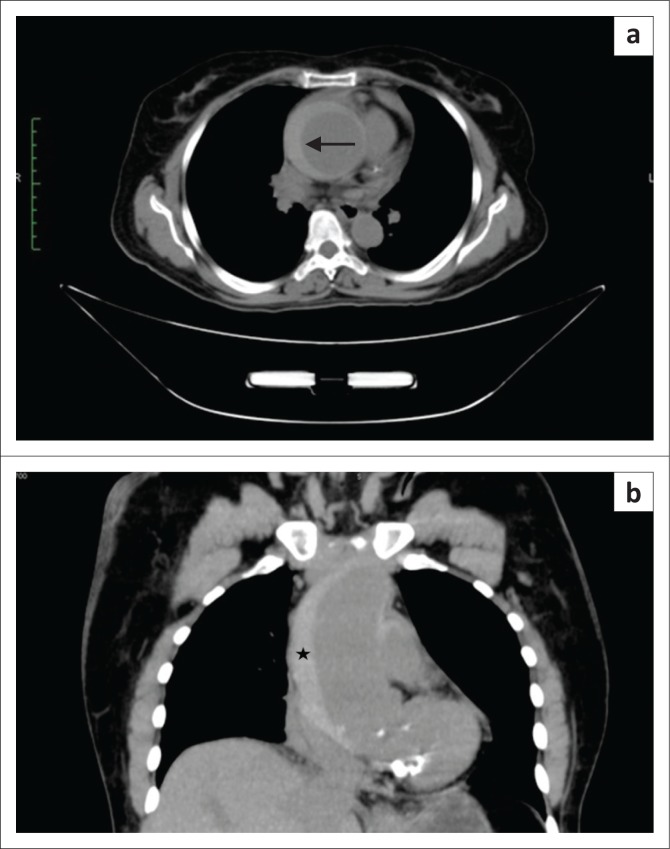
Stanford type A intramural haematoma (IMH): (a) Unenhanced axial computed tomography (CT) scan showing hyperdense crescentic aortic wall thickening involving the ascending aorta (arrow); (b) Stanford type A IMH. Unenhanced coronal CT scan showing crescentic hyperdense aortic wall thickening (*) compressing the lumen.

**FIGURE 7:**
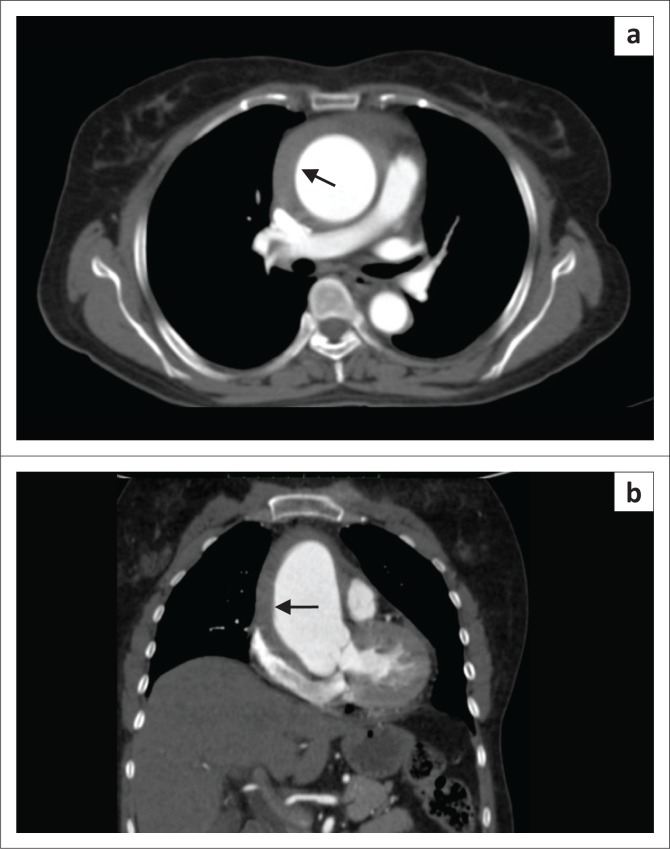
Type A intramural haematoma. Contrast-enhanced axial (a) and coronal (b) computed tomography scan showing a non-enhancing hypodense crescent-shaped area (arrow) compressing the normal contrast-enhanced aortic lumen.

**FIGURE 8:**
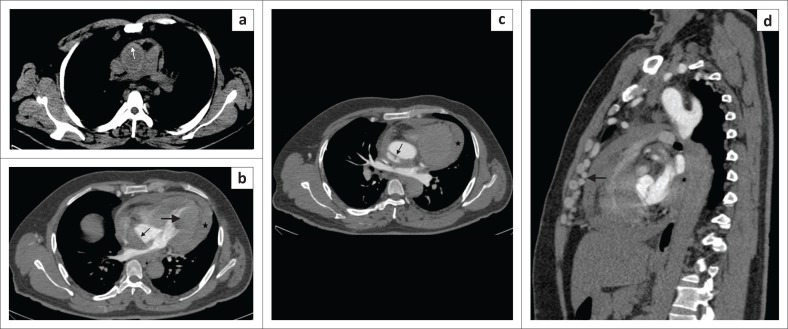
Coarctation of the aorta with a type A intramural haematoma (IMH) and aortic dissection. (a) Unenhanced axial computed tomography (CT) scan showing circumferential hyperdense thickening of the ascending aorta (arrow); (b) Contrast-enhanced axial CT showing crescentic non-enhancing hypodense area (thin arrow) representing IMH with haemopericardium (*) and concentric left ventricular hypertrophy (thick arrow); (c) Contrast-enhanced CT scan showing the displaced intimal flap (arrow) and haemopericardium (*). (d) Sudden narrowing of the aorta (arrow) distal to origin of the left subclavian artery on sagittal contrast-enhanced images. Multiple collaterals (thick arrow) along the anterior chest wall.

Crescentic aortic wall thickening of increased attenuation is the most vital finding on unenhanced CT scan images (Figures 6 and 8a). The lesion will displace intimal calcifications internally, if present. On contrast-enhanced images, the aortic lumen appears narrow without enhancement of the lesion (Figures 7 and 8b). On contrast-enhanced CT, it is difficult to distinguish between a thrombosed false lumen of AD and IMH. Aortic dissection follows a spiral longitudinal course, whereas IMH has a constant circumferential relation to the aortic wall which may aid in differentiation.^11^

Management of IMH is similar to AD, where surgery is advised for type A lesions and type B IMHs are managed conservatively.^12^

## Penetrating atherosclerotic ulcer

Penetrating atherosclerotic ulcer is the ulceration of atheromatous plaque, resulting in disruption of the internal elastic lamina and aortic media with haematoma formation. Penetrating atherosclerotic ulcer typically occurs in the elderly with a background of advanced atherosclerosis, in contrast to AD which is seen in younger patients. The incidence of PAU in AAS ranges between 2.3% and 7.6%.^13^ The aortic arch and descending aorta are the most common sites. Clinically, the symptoms of PAU are similar to those seen in AAS.

### Imaging findings

**FIGURE 9:**
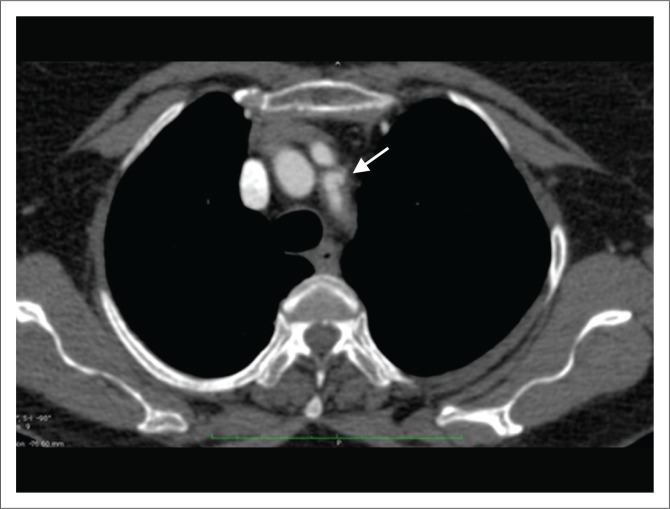
Penetrating atherosclerotic ulcer. Contrast-filled outpouching seen beyond the aortic wall involving the arch of the aorta (arrow).

**FIGURE 10:**
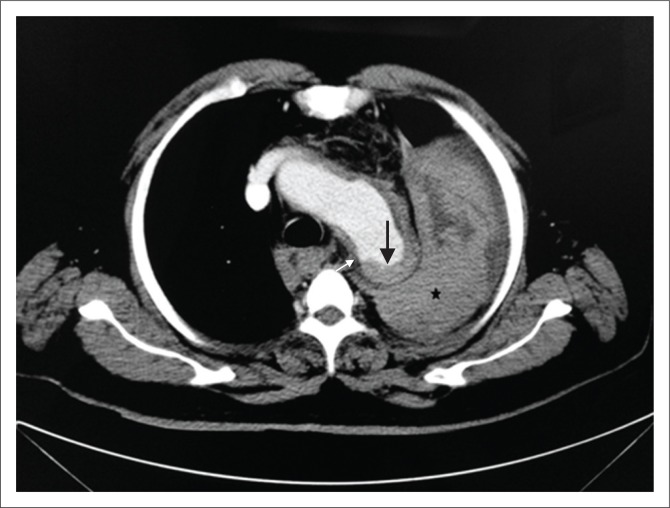
Ruptured penetrating atherosclerotic ulcer (PAU) with contained haematoma. Contrast-enhanced computed tomography scan of thorax showing a ruptured PAU along the posterior aortic wall (white arrow) with adjacent intramural haematoma (black arrow) appearing as hyperdense aortic wall thickening and a contained haematoma in left pleural space (*). Findings were confirmed at surgery.

Extensive intimal calcifications are the frequent finding on unenhanced CT. A contrast-filled outpouching extending beyond the aortic wall is diagnostic of PAU (Figure 9). Hyperdense haematoma adjacent to the ulceration may be seen on unenhanced CT.^14^ The lesions can be single or multiple. Adjacent aortic wall thickening with atheromatous plaques are often seen. Penetrating atherosclerotic ulcer can progress to form a saccular aneurysm or even rarely, rupture. Additional imaging findings like haemothorax, haemopericardium, mediastinal haematoma, AD and ischemic findings owing to vascular obstructions can be seen as complications of PAU (Figure 10).

Medical management is the preferred treatment option for type B lesions, as in AD, with follow-up imaging to observe progression. Surgery is the treatment of choice for patients with aortic rupture, persistent pain, haemodynamic instability and risk of embolisation. The disease prognosis remains poor as surgical intervention is difficult owing to extensive atherosclerotic changes in the remaining aorta.

## Conclusion

A dedicated CT protocol comprising an initial unenhanced study followed by post-contrast imaging should be immediately performed in patients suspected with AAS. Knowledge about the imaging findings of these interrelated conditions can facilitate prompt diagnosis.
